# Interplay of Heme with Macrophages in Homeostasis and Inflammation

**DOI:** 10.3390/ijms21030740

**Published:** 2020-01-23

**Authors:** Pooja Pradhan, Vijith Vijayan, Faikah Gueler, Stephan Immenschuh

**Affiliations:** 1 Institute for Transfusion Medicine, Hannover Medical School, Hannover 30625, Germany; pradhan.pooja@mh-hannover.de (P.P.); vijayan.vijith@mh-hannover.de (V.V.); 2 Department of Nephrology, Hannover Medical School, Hannover 30625, Germany; gueler.faikah@mh-hannover.de

**Keywords:** BACH1, heme, heme-binding proteins, inflammation, macrophages

## Abstract

Macrophages are an integral part of the mononuclear phagocyte system that is critical for maintaining immune homeostasis. They play a key role for initiation and modulation of immunological responses in inflammation and infection. Moreover, macrophages exhibit a wide spectrum of tissue-specific phenotypes in steady-state and pathophysiological conditions. Recent clinical and experimental evidence indicates that the ubiquitous compound heme is a crucial regulator of these cells, e.g., in the differentiation of monocytes to tissue-resident macrophages and/ or in activation by inflammatory stimuli. Notably, heme, an iron containing tetrapyrrole, is essential as a prosthetic group of hemoproteins (e.g., hemoglobin and cytochromes), whereas non-protein bound free or labile heme can be harmful via pro-oxidant, pro-inflammatory, and cytotoxic effects. In this review, it will be discussed how the complex interplay of heme with macrophages regulates homeostasis and inflammation via modulating macrophage inflammatory characteristics and/ or hematopoiesis. A particular focus will be the distinct roles of intra- and extracellular labile heme and the regulation of its availability by heme-binding proteins. Finally, it will be addressed how heme modulates macrophage functions via specific transcriptional factors, in particular the nuclear repressor BTB and CNC homologue (BACH)1 and Spi-C.

## 1. Introduction

Macrophages, white blood cells named after their ability to phagocytose, belong to the mononuclear phagocyte system and play a vital role in maintaining immune homeostasis by ingesting and killing pathogens, as well as clearing up dead cells and debris [1]. In addition to their role in immune surveillance, macrophages exhibit heterogeneous organ-specific physiological functions, which are induced and maintained by tissue-specific environmental factors [2]. For example, retinoic acid-mediated signaling in peritoneal macrophages regulates the local response of B1-cells, a subpopulation of B-cells involved in gut humoral immunity [3], receptor activator of NF-κB ligand (RANK-L) modulates signaling in osteoclasts to regulate osteoblast function [4], and granulocyte macrophage colony-stimulating factor (GM-CSF) mediates differentiation of alveolar macrophages in the lung [5]. Notably, the tetrapyrrole heme has been recognized as a micro-environmental factor for the differentiation and maintenance of red-pulp macrophages (RPMs) in the spleen [6]. In addition, heme has also been shown to affect the inflammatory phenotypes of macrophages in a seemingly contradictory manner.

Heme, a central iron-containing tetrapyrrole, is critical for sustaining life in aerobic organisms. It is the prosthetic group of numerous hemoproteins and is essential for various biological functions such as oxygen storage and transport, drug metabolism, or electron transfer in the respiratory chain [7]. In contrast, ‘free’ heme can catalyze the Fenton reaction due to its reactive iron, which makes this molecule potentially toxic [8]. Hence, it is crucial that heme levels are tightly regulated. For example, the majority of heme synthesis occurs in immature red blood cells (RBCs) and only limited heme synthesis occurs in non-erythroid cells [9]. Additionally, heme induces its own synthesis in erythroid cells, but inhibits its synthesis in non-erythroid cells. Moreover, cells have heme-degrading enzymes available such as heme oxygenase (HO) to control intracellular heme levels. Two isoforms of HO are known: a constitutive isoform termed HO-2, and an inducible isoform termed HO-1 [10,11]. In physiological and pathophysiological conditions expression of HO-1 is highest in macrophages [12,13,14,15,16]. A major function of the heme-HO-1 system in the myeloid-mononuclear system appears to be the control of erythropoiesis [17]. Moreover, in pathophysiological conditions, this complex system is critical for regulating the inflammatory phenotype of these cells [18]. 

## 2. The Intra- and Extracellular Pool of Labile Heme

The major fraction of total heme is covalently or non-covalently bound as a prosthetic group in hemoproteins (e.g., myoglobin, cytochromes, and hemoglobin (Hb)) [7,19]. In the current review we mainly focus on the so-called ‘labile’ heme pool, also termed regulatory or bioavailable heme pool, which represents a minor fraction of total cellular heme [20,21,22,23] and appears to be of major significance for governing multiple regulatory pathways. Labile heme has been proposed to be available in a ‘loosely bound’ state that makes it readily exchangeable between proteins and can be further categorized into intra- and extracellular heme fractions.

Intracellular labile heme concentrations in steady-state conditions have been suggested to be in the range of 20 to 340 nM [24]. Independent studies have indicated the presence of labile heme in different cellular compartments such as the nucleus, mitochondria, cytosol, Golgi complex, and endoplasmic reticulum [23,25], leading to the notion that this heme fraction might have intracellular signaling functions. Accordingly, we have shown that the regulatory pattern of labile heme levels correlated with that of HO-1 in macrophages [26]. Specifically, in mouse macrophages, stimulation with the TLR4 ligand lipopolysaccharide up-regulated intracellular labile heme levels and HO-1, whereas in human macrophages, the same stimulus led to an opposing regulatory pattern [26]. Thus, we hypothesize that labile heme is an up-stream signal required for HO-1 regulation and may function via interaction with the heme-sensitive transcriptional factor (TF) BTB and CNC homologue (BACH1), which will be discussed in detail below. The intracellular labile heme pool might also be used for incorporation into high turnover proteins such as cyclooxygenase-2, NADPH oxidase-2, and inducible nitric oxide synthase (iNOS), all of which are up-regulated by pro-inflammatory stimuli in immune cells such as macrophages to mount an efficient inflammatory response. It is conceivable, that the labile heme pool is controlled via the enzymatic synthesis of heme in cellular steady-state because its inhibition either by succinylacetone or N-methyl protoporphyrin IX down-regulates intracellular heme levels [20,21,26]. Notably, labile heme does not seem to be degraded by up-regulation of the heme-degrading enzyme HO-1, which might be explained by the K_m_ of HO-1 for heme (<1 µM) [27,28]. Although our understanding on the functional role of the intracellular labile heme pool in non-erythroid cells is limited and details are only emerging, various recently developed methods [23,25,29] for determining labile heme might help to further characterize and substantiate the role of heme as an intracellular signaling molecule. It will also be interesting to explore potential cell-type specific differences of labile heme levels, which might determine their functional phenotype.

The term labile heme has also been used in the context of extracellular heme which can occur in clinical conditions such as hemolysis and/or tissue injury [30,31,32,33]. In such pathophysiological settings the source of labile heme appears to be mainly damaged erythrocytes, myoglobin or heme released from hemoproteins. In contrast to intracellular labile heme, extracellular labile heme has been proposed to be a damage/danger-associated molecular pattern (DAMP) [30,31,32,33].

## 3. Role of Extra- and Intra-Cellular Heme-Binding Proteins (HBPs) in the Regulation of Labile Heme Homeostasis

HBPs are non-hemoproteins that can reversibly bind heme and thereby modulate heme properties. Two important functions of HBPs appear to be the protection against heme toxicity via neutralizing its pro-oxidant properties and/ or via controlling heme bioavailability. 

### 3.1. Extracellular HBPs Neutralize Labile Heme

HBPs are abundant in blood vessels as serum proteins and ensure that labile heme released via oxidation of extracellular Hb during hemolysis [34,35] is neutralized in a time-dependent manner. Heme bound by HBPs may affect the pro-oxidant capacity depending on the binding affinity of the respective protein [36,37]. Moreover, the binding of heme by HBPs may block the unspecific uptake of heme into cells such as endothelial cells, thereby limiting their leakage into the extravascular space [35] (Table 1). Two well-known serum HBPs are hemopexin (Hx) [38] and albumin [39]. Hx binds heme with the highest binding affinity of all known proteins and transports heme to the liver for degradation [40], whereas albumin, due to its abundance, might act as a transient HBP and transfer heme to Hx in a step-wise process. Other serum HBPs are α1-microglobulin [41] and α1-antitrypsin [42], but their respective physiological roles as HBPs are not well characterized (Table 1).

### 3.2. Intracellular HBPs Regulate Bioavailability of Labile Heme 

Several intracellular HBPs have been identified [24,45] as heme transporters in different subcellular compartments (Table 2). For example, the HBP heme responsive gene (HRG)-1 protein has been shown to transport heme from the phagolysosomal compartment to the cytosol after erythrophagocytosis in macrophages [46]. Similarly, upon CD163-mediated endocytosis, heme carrier protein (HCP)-1 was found to co-localize with Hb/haptoglobin (Hp) complexes in early endosomes of human macrophages [47], suggesting that HCP-1, similar to HRG-1, may be a functional heme transporter from lysosomes to the cytosol. These proteins might also play a role in heme transport from lysosomes after degradation of intracellular hemoproteins. On the other hand, the HBP Feline leukemia virus subgroup C receptor 1a (FLVCR 1a) is a cell surface heme exporter and might play a role in macrophages based on the observation that mice with genetic deficiency for FLVCR1 are impaired in iron reutilization [48,49]. The need for cells to import heme from the extracellular space has been controversially discussed given that the cells have their own heme synthesis and degradation machinery [27]. However, it is likely that these transporters play a critical regulatory role for heme homeostasis in macrophages due to their known role in heme acquisition.

Notably, several proteins have been identified as putative heme chaperones that may mediate the intracellular trafficking of labile heme and/or provide a buffering environment to ensure that heme does not cause cytotoxicity [24,60]. Interestingly, some of the known heme chaperones are proteins that are recognized to exhibit various other biological functions. For example, the well-known glycolytic enzyme glyceraldehyde phosphate dehydrogenase (GAPDH) facilitates the incorporation of heme into hemoproteins such as iNOS and Hap1 as a heme chaperone [22,25,55]. More recently, the progesterone receptor membrane component 2 (PGRMC2) has been shown to be important for delivering labile heme to the nucleus in adipocytes [61]. Other examples of heme chaperones are peroxiredoxin-1 synonymous with heme-binding protein 23 (HBP23) [58,62], biliverdin reductase (BVR) [53], glutathione-S-transferases [63], fatty acid binding protein [54], all of which have been shown to bind heme. However, a conclusive role for these proteins in intracellular heme trafficking is yet to be demonstrated [24]. In conclusion, it appears that the bioavailability of intracellular heme is determined by a complex network of regulatory mechanisms involving several intracellular heme transporters and heme chaperones. Whether these various transporters and chaperones work in coordination to control the intracellular heme dynamics is only incompletely understood and currently under investigation. 

### 3.3. Intracellular Labile Heme Controls Cellular Functions via Heme-Sensor Proteins

**Figure 1:**
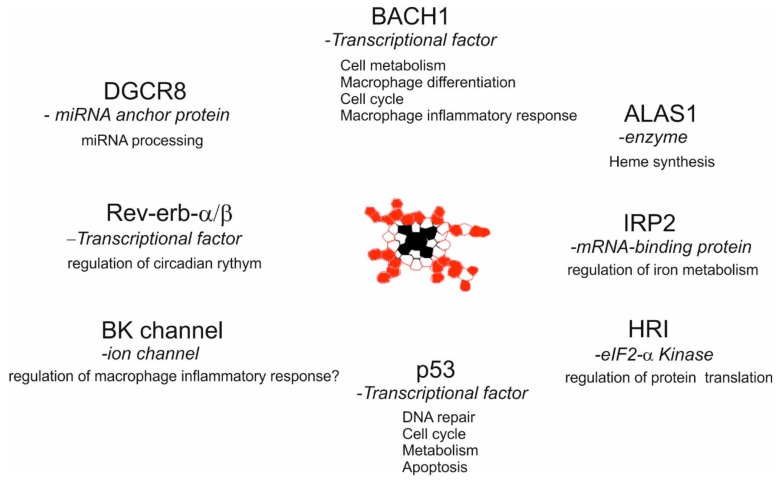
**Heme-mediated control of cellular functions via heme sensor proteins.** Intracellular labile heme may control various aspects of macrophage functions via binding and modulation of the indicated proteins through their heme regulatory motifs (CP/CXXH/PC). Iron regulatory protein 2 (IRP2);5’-Amino levulinate synthase-1 (ALAS1); Heme regulated eIF2-a kinase (HRI); DiGeorge syndrome critical region 8 (DGCR8); Big potassium (BK).

Apart from heme transporters and chaperones, there are intracellular HBPs classified as heme sensor proteins which are regulated upon the binding of heme, and in turn modulate central cellular functions. One such classical heme sensor protein is the TF BACH1 [64]. The interaction of BACH1 with heme via its heme-binding site results in proteasome-dependent degradation of this protein [65], and the subsequent activation of genes which are repressed by BACH1. Although, BACH1 has initially been identified as a transcriptional regulator of HO-1 [66], recent studies have indicated that it controls various aspects of cellular function such as bioenergetics [67], cell cycle [68], and macrophage differentiation [69]. Specifically, in monocytes BACH1 was also shown to be involved in the subtype specification of lymphocyte antigen 6C (Ly6C)+ (inflammatory) and Ly6C- (patrolling) monocytes, the underlying mechanisms of which are yet to be characterized [70]. Similarly, the binding of heme to the nuclear orphan receptors rev-erb-α/β represses the expression of their target genes implicating heme to be a signaling molecule for the regulation of the circadian rhythm [71,72]. Furthermore, heme has also been shown to bind and degrade the tumor suppressor p53 [73], which is critical for a variety of cellular pathways, such as regulation of the cell cycle, cell death, DNA-damage repair, and metabolism [74,75]. The various biological functions that can be influenced by heme interactions with heme sensor proteins are summarized in Figure 1. Collectively, these findings suggest that labile heme plays a key role in intracellular signaling and interacts with specific HBPs, the functionality of which is beyond its classical role as a prosthetic group in hemoproteins. 

## 4. How Heme and Macrophages Control Erythropoiesis in Steady State and during Inflammation?

In mammals, heme and macrophages have formed complex mutual regulatory interactions. Heme is involved in the differentiation of tissue-specific macrophages that are involved in RBC degradation and recycling of this compound. Moreover, the interplay of heme with macrophages has intriguing regulatory effects in inflammatory activation.

### Heme Sustains Erythropoiesis through Differentiation of Erythrophagocytosing Macrophages 

Specialized macrophage functions appear to play a pivotal role in controlling erythropoiesis. Senescent RBCs are removed from the circulation via RPMs in the spleen, and to a lesser extent by Kupffer cells in the liver [17]. These cells are characterized by high constitutive HO-1 expression, which may facilitate their tolerance against high levels of intracellular heme. Heme degradation by HO-1, in turn recycles the iron which is exported out of the cell via ferroportin and eventually is used for de novo erythropoiesis [76,77]. Moreover, it has been demonstrated that the heme-dependent induction of the TF Spi-C is responsible for the differentiation of these erythrophagocytosing macrophages [6]. The crucial role of heme in the differentiation of erythrophagocytosing macrophages is further underscored by the findings that heme-mediated Spi-C induction via BACH1 degradation in bone marrow monocytes renders them with a pre-erythrophagocytosing phenotype [6], and bone-marrow transplantation in HO-1-/- mice replenishes the erythrophagocytosing macrophage population in the liver [77]. Similarly, a recent study showed that central macrophages in erythroblastic islands of the bone marrow involved in nourishing and supporting erythroid maturation are also characterized by a high expression of Spi-C and iron recycling genes such as HO-1 [78] which suggests a possible role for heme in the differentiation of these macrophages. It is important to note that Spi-C has no established heme-binding motifs and heme-dependent induction of Spi-C at least in monocytes is mediated via the de-repression due to degradation of BACH1 [6]. In pathophysiological situations such as chronic inflammatory disorders the erythrophagocytosing properties of macrophages may also contribute to the anemia of inflammation (AI). AI may be considered as a host defense strategy during infectious diseases ensuring that pathogens have limited access to iron. During inflammation or infection, erythrophagocytosis-mediated heme acquisition by macrophages leads to the expression of ferritin, which safeguards iron inside the cell. Additionally, macrophage or hepatocyte secreted hepcidin binds to ferroportin and promotes its internalization and degradation to prevent further iron export [79]. In this way, down-regulation of iron export contributes to a decrease in erythropoiesis. Notably, in vitro LPS and interferon γ-mediated (pro-inflammatory) polarization of human macrophages leads to an iron-retention phenotype characterized by the low expression of ferroportin and high expression of ferritin. By contrast, IL-4-mediated (anti-inflammatory) polarization leads to an iron-release phenotype characterized by high expression of ferroportin and low expression of ferritin indicating that the inflammatory status of the cell also influences its iron-regulatory nature [80]. Whether the differential expression patterns of iron-regulatory genes are affected by intracellular labile heme levels remains to be evaluated. On the other hand, erythrophagocytosis by spleen macrophages has been implicated in the induction of stress-erythropoiesis in this organ. Heme-driven Spi-C induction in these macrophages leads to the expression of bone morphogenic protein 4 and growth and differentiation factor 15, which in turn leads to the expansion of stress erythroid progenitors in the spleen to sustain erythropoiesis to account for the loss of steady-state erythropoiesis [81]. Collectively, the existing literature supports the notion that heme controls its own demand for erythropoiesis partially through macrophages during homeostasis and inflammation.

Interestingly, heme also appears to control up-stream hematopoiesis. It has recently been shown that the heme-regulated TFs BACH1 and 2 are critical for controlling homeostasis of erythro- and myelopoiesis via modulation of lineage commitment in hematopoietic stem and progenitor cells (HSPCs). In particular, these TFs are critically controlling erythropoiesis under steady-state and stress conditions as demonstrated in a BACH1 and 2 double knockout mouse model [82]. Specifically, it has been shown that BACH1 and 2 are down-regulated in HSPCs in inflammatory conditions and, in turn, promote myeloid differentiation at the expense of erythropoiesis. An important role in this regulatory pathway has been ascribed to the mutual interaction of BACH1 and 2 with the pro-inflammatory TF C/EBPbeta at the activating protein-1 (AP-1) binding site within the BACH recognition element [82]. Notably, BACH1 and 2-dependent modulation of hematopoiesis at the erythro-myeloid bifurcation may not only be important in the pathogenesis of AI, but also in that of the myelodysplastic syndrome [83,84]. The detailed role of heme in these complex regulatory pathways of HSPCs remains to be established.

## 5. Role of Heme in Inflammatory Activation of Macrophages

A more controversial role of heme has emerged with respect to its potential in regulating inflammatory responses in macrophages. Heme has been shown to polarize macrophages either to induce a pro-inflammatory response (also known as M1) or to induce an anti-inflammatory response (also known as M2). The pro-inflammatory response facilitates the neutralization and elimination of pathogens, whereas the anti-inflammatory response of macrophages is involved in tissue repair after inflammation. 

### 5.1. Heme as an Anti-Inflammatory Signal in Macrophages

CD163 is a cell-type specific surface receptor of macrophages which mediates the up-take of Hb/Hp complexes and is also a well-established marker for anti-inflammatory (M2) macrophages. It has been demonstrated that the uptake of Hb-heme via CD163 induces HO-1 and ferritin expression in macrophages to confer an anti-inflammatory protective phenotype [85]. Similarly, this mechanism may also elicit the secretion of the anti-inflammatory cytokine IL-10 [86]. The protective role of Hb uptake in macrophages was further observed in vivo in post-hemorrhage settings [87,88]. Importantly, heme may induce an anti-inflammatory response in macrophages via HO-1-dependent heme catabolism. The catalytic breakdown of heme by HO-1 generates the products iron, carbon monoxide (CO) and biliverdin, the latter of which is converted to bilirubin. Both CO and bilirubin have potent anti-inflammatory and antioxidant properties, respectively, the mechanistic details of which are reviewed in detail previously [89,90]. Heme-mediated anti-inflammatory macrophage reprogramming has also been demonstrated in an animal model of pancreatitis. Administration of heme and subsequent induction of HO-1 in peritoneal macrophages alleviated inflammation in a choline-deficient diet mouse model of pancreatitis [91]. Similarly, peritoneal administration of heme was found to protect against experimental colitis via up-regulation of Spi-C in macrophages [92]. The anti-inflammatory effects of Spi-C are mediated by its association with the interferon regulatory factor (IRF)5, thereby blocking the interaction of IRF5 with the NF-kB subunit p65, which is required for the transcriptional regulation of pro-inflammatory genes such as IL-6 and IL-1α. Similar to RPMs, intestinal macrophages also exhibit high basal levels of Spi-C suggesting that heme might also be a functional demand signal required for the intestinal macrophage phenotype in physiological conditions [92]. This might further contribute to the non-inflammatory status of these cells which may allow their co-existence with the gut microbiome. The potential role of BACH1 for heme-mediated Spi-C induction in intestinal macrophages was not evaluated in this study. Independently, heme encapsulated in liposomes has been shown to target macrophages at the site of injury after myocardial infarction and to reprogram their inflammatory nature to an anti-inflammatory phenotype [93]. In summary, the administration of heme in vivo has been shown to cause anti-inflammatory polarization of macrophages which might be mediated via BACH-1 degradation and subsequent induction of HO-1 and Spi-C.

### 5.2. Heme as a Pro-Inflammatory Signal in Macrophages

In contrast to the above described findings, several in vitro studies have demonstrated that heme may cause pro-inflammatory activation of macrophages. Heme appears to act as a pro-inflammatory second hit in macrophages and aggravates LPS-induced TLR4 signaling, leading to the increased secretion of pro-inflammatory cytokines such as TNF-α and IL-6, and also acts as a second signal for inflammasome activation [94,95,96]. Furthermore, the pro-inflammatory effects of heme were not observed in the absence of serum indicating additional unknown serum factors to be involved in this phenomenon. Intriguingly, it has been reported that heme is not, per se, an inducer of TLR4 signaling and the presence of serum or heme prepared with albumin blocks the pro-inflammatory effects associated with heme [97]. The lack of heme-induced pro-inflammatory effects was not due to blockage of heme up-take because heme-albumin complexes readily induced HO-1 expression and Spi-C in these macrophages [97]. The discrepancy between these in vitro findings raises various questions such as: (1) the clinically relevant pathophysiological heme concentration to be used in in vitro settings; (2) the physiological relevance of in vitro used heme preparations without albumin; (3) how do macrophages take up heme in these different settings? Additionally, it needs to be considered that, due to its hydrophobicity, heme might readily intercalate within the lipid bilayer and induce lipid peroxidation, which can further cause a non-specific stress activation in these cells. Hence, in vitro studies with heme preparations in macrophages should be scrutinized and categorized in more detail to better understand when extracellular heme may become pro-inflammatory. It appears to be unlikely that, in the intravascular space, heme might exist in a non-albumin bound form due to the abundance of albumin. By contrast, it is conceivable that heme, which may be present in the extravascular space due to tissue injury or cell damage might be present in a non-albumin-bound form and could act as a DAMP (second hit) to aggravate an inflammatory response. Accordingly, studies from Nath and colleagues have shown a detrimental role of heme released from hemoproteins in kidney injury [98,99,100]. Furthermore, in an experimental model of renal ischemia-reperfusion injury (IRI), we recently detected high levels of local labile heme in kidneys, which was dependent on the IRI-time and correlated with the IRI-mediated inflammation [101]. The source of heme in this setting is currently not clear but indicates that extracellular labile heme might aggravate an inflammatory response [101]. Alternatively, labile heme levels in the intravascular space might mediate inflammatory effects on macrophages via secondary factors such as complement activation [102,103]. Accordingly, a recent report indicated that stimulation of whole blood with heme activates cytokine secretion in leukocytes via complement activation [104]. The mechanisms of how labile heme might be involved in the pathogenesis of inflammatory disorders are not well understood. Studies in HO-1 knock out mouse models do not seem to be well-suited to investigate a specific regulatory role of heme, because HO-1 is a cytoprotective gene and it is conceivable that loss of HO-1 makes these models highly sensitive to general stress stimuli. Moreover, the injection of heme and/ or reduction of serum levels of HBPs have been used as an experimental approach to study the role of heme in inflammatory conditions. These strategies, however, only provide indirect evidence and do not distinguish the functionality, source and localization of labile heme.

The contrasting role of labile heme in pathophysiological conditions is also depicted in sepsis. Larsen et al. demonstrated that extracellular labile heme may act as a DAMP [105]. In contrast, others have proposed hemophagoctosis and subsequent HO-1 expression as a protective mechanism in sepsis patients [16]. Intriguingly, in a recent report using a systems biology approach, it was observed that the surviving sepsis patients had elevated expressions of heme synthesis genes and iron-recycling genes in the white blood cells, suggesting a protective role for the heme-HO-1 pathway [106]. These observations further substantiate the need to further investigate the effects of heme in a context- and cell type-specific manner to understand its contradictory roles.

## 6. Conclusions and Outlook 

The interplay between heme and macrophages plays a critical role in homeostasis and inflammation. Emerging evidence indicates that intracellular labile heme is a key signaling molecule for a number of regulatory pathways. The functions of labile heme are determined by its availability via interactions with intra- and extracellular HBPs. In macrophages the heme-responsive TFs BACH1 and Spi-C appear to be of major functional significance. Moreover, the role of extracellular heme in macrophage polarization and inflammatory activation appears to be ambiguous. Further characterization on the underlying mechanisms of heme acquisition by macrophages in pathological conditions will help to better understand the heme-bestowed inflammatory status of macrophages, and to ultimately afford novel therapeutic strategies.

## Figures and Tables

**Table 1:** Extracellular heme binding proteins in mammalians.

Heme Binding Protein	Heme Affinity (Kd) (M)	Serum Concentration	Ref.
Hemopexin	1 × 10^−14^	0.6–1.2 g/L	[38,43]
Albumin	1.2 × 10^−8^	35–53 g/L	[39,44]
α1-Microglobulin	1 × 10^−6^	0.03 g/L	[41]
α1-Antitrypsin	2 × 10^−8^	1.3–2.5 g/L	[42]

**Table 2:** Intracellular heme binding proteins in mammalians.

Heme binding protein	Function	Ref
***Putative heme transporters***		
Feline leukemia virus subgroup C receptor 1a (FLVCR1a)	export of heme to extracellular space	[48,50]
Feline leukemia virus subgroup C receptor 1a (FLVCR1b)	export of heme from the mitochondria to cytosol	[51]
Feline leukemia virus subgroup C receptor 2 (FLVCR2)	import of heme from extracellular space	[52]
Heme responsive gene-1 (HRG-1)	export of heme from phagolysosome to cytosol	[46]
Heme carrier protein-1 (HCP-1)	export of heme from lysosome to cytosol (?)	[47]
***Putative heme chaperones***		
Biliverdin reductase (BVR)	heme trafficking to nucleus (?)	[53]
Fatty acid binding protein (FABP)	heme trafficking in cytosol (?)	[54]
Glceraldehyde phosphate dehydrogenase (GAPDH)	heme trafficking in cytosol	[22], [55]
GSH-S-transferase	heme trafficking in cytosol (?)	[56]
Heme binding protein 22 (HBP22)	heme trafficking in cytosol (?)	[57]
Heme binding protein 23 (HBP23)	heme trafficking in cytosol (?)	[58]
SOUL	heme trafficking in cytosol (?)	[59]
